# Co-requirement for B cells to protect against pulmonary wild-type *Brucella melitensis* challenge following mucosal vaccination with a live *Brucella* vaccine prototype

**DOI:** 10.3389/fimmu.2026.1925725

**Published:** 2026-10-23

**Authors:** Zakia I. Goodwin, Carol Hoffman, Ella Bhagyaraj, David W. Pascual

**Affiliations:** 1 Department of Infectious Diseases & Immunology, University of Florida College of Veterinary Medicine, Gainesville, FL, United States; 2 Department of Veterinary Science, College of Agriculture, Life Sciences, & Natural Resources, University of Wyoming, Laramie, WY, United States

**Keywords:** B cells, *Brucella*, nasal immunization, T cells, vaccines

## Abstract

Mice nasally vaccinated with the *Brucella melitensis* (BM) double-mutant (Δ*znuA* Δ*norD B. melitensis*-mCherry [znBM-mC]) showed increased numbers of B cells residing in the lungs 5 days post-vaccination and contributing to antigen-presentation. Studies were pursued to determine the relevance of B cells in protection conferred by mucosal znBM-mC vaccination of BALB/c compared to B cell null (µMT) mice. Two vaccination regimens were applied: oral prime, nasal boost versus a single nasal vaccination. Both methods have proven efficacious in immunocompetent mice. BALB/c mice, but not µMT mice, vaccinated with znBM-mC showed the expected efficacy. Robust T cell recruitment and differentiation were observed in the lungs in both mouse strains with no significant differences in total CD4^+^ and CD8^+^ T cells post-vaccination, although some subset phenotypic differences were noted in µMT mice. Specifically, more proinflammatory T cells were present in µMT lungs 4 weeks post-boost evident by increased numbers of TNF-α^+^ CD4^+^ and CD8^+^ T cells, polyfunctional CD4^+^ and CD8^+^ T cells, and IFN-γ^+^ CD8^+^ T cells. Examination of lung memory T cells showed no differences between vaccinated mouse groups in numbers of resident memory T cells (TRMs), central memory T cells (TCMs), and CD8^+^ effector memory T cells (TEMs); the CD4^+^ TEMs were slightly reduced in µMT lungs. Adoptive transfer of naive or immune B cells prior to wild-type (wt) BM 16M challenge in vaccinated µMT mice enabled protection. In the context of a vaccine-induced response, adoptive transfer of immune B cells into unvaccinated µMT mice failed to protect against wt BM 16M challenge demonstrating that znBM-induced protection involves both B and T cell contributions. In addition to triggering recruitment and expansion of proinflammatory and memory T cell subsets, mucosal vaccination recruits a population of B cells, which enhance protection to challenge.

## 1 Introduction

While primarily a disease of livestock resulting in fetal abortion (1, 2), brucellosis in humans is a significant global health concern (3–6), ranking as the third most neglected zoonotic disease worldwide (7). The high incidence of brucellosis (5, 8), which is believed to be 2 million new cases annually (9), is linked to the prevalence of disease in infected livestock (10, 11), the source of unpasteurized milk commonly used in dairy products (12–16). Oral exposure remains the primary route of human infection (13, 17, 18), even in the US (1, 12, 15). Since there are no vaccines available for humans, current treatment uses a complex, two-antibiotic regimen for six weeks (14), which does not guarantee clearance of brucellae leaving 16% of patients infected, and often leads to long-term chronic disease (4, 5, 10).

While live, attenuated vaccines are available for livestock, none exists for humans. Their efficacy in livestock is estimated to be about 70% (1, 2), warranting the development of new vaccines. Improving vaccine performance has met with limited success (1, 2, 18). *Brucella* infections primarily occur via a mucosal exposure (1, 5, 8, 12), yet few studies have explored vaccination by the mucosal route (1). The absence of brucellae or pathology in the intestinal tract (19, 20) of nonhuman primates (21) and in humans (17, 22), suggests that other mucosal sites, such as oro-pharyngeal tissues, may serve as primary entry points for humans (1, 23–26). In support of this, studies with wild-type *B. melitensis* (wt BM) have shown sustained infection of the cervical lymph nodes following oral infection (23, 25). In mice, oral infection with wt *B. abortus* (BA) can persist in the systemic (splenic) compartment for > 20 wks (27). To test for the relevance of mucosal vaccination, we have achieved exquisite protection against pulmonary BM 16M challenge following a single oral or nasal dose of Δ*znuA* BM (28, 29) or with two doses of the double-mutants, Δ*znuA* Δ*norD B. abortus-lacZ* (znBAZ; 30) or Δ*znuA* Δ*norD B. melitensis*-mCherry (znBM-mC; 31). In contrast, intranasal (IN) immunization with conventional RB51 vaccine has proven to be poorly protective against pulmonary *Brucella* challenge (32–34).

Protection against brucellosis is primarily T cell-dependent, driven by IFN-γ (1, 18), and such results were mostly derived from parenteral vaccination studies. However, protection against pulmonary challenge with virulent *Brucella* was found to be dependent upon vaccine-induced lung IFN-γ- and TNF-α-producing resident memory CD8^+^ T cells (29–32), since efficacy was abated in CD8-deficient mice (29, 30). In contrast, protection remained unabated in either znBM-mC-vaccinated CD4- or CD8-deficient mice suggesting that either memory CD4^+^ or CD8^+^ T cells contributed to protection against wt BM 16M (31). The role of B cells in protection to brucellosis has long been debated and believed not necessary for protection (1, 18). B cells are thought to play a lesser role in *Brucella* infections, as antibodies (Abs) do not confer protection (18, 35). Often not considered are B cells’ other functions unrelated to Ab production. Interestingly, B cells have a phagocytic capacity (36), particularly B1 cells (37, 38). Along these lines, B cells were found to be highly susceptible to infection by brucellae (39, 40), eliciting regulatory cytokines (39, 41). Both *in-vitro* and *in-vivo* studies indicated that BA infection via IgM and complement opsonization occurs in B cells, with the *Brucella* surviving, though not replicating within these cells (39).

A notable B cell influx into the lungs following a single nasal dose of znBM-mC was observed, suggesting that B cells were important for protection mediated by znBM-mC. Vaccination of µMT mice showed a lack of efficacy using znBM-mC, unless adoptive transfer of naïve or immune B cells into µMT mice enabled protection.

## 2 Materials and methods

### 2.1 *Brucella* strains

The double mutant, attenuated znBM-mC and znBM-lacZ (znBMZ) were derived from wild-type (wt) BM 16M as previously described (31). Both znBM-mC and wt BM 16M were propagated on Potato Infusion Agar (PIA) plates at 37 °C and 5% CO_2_ for 3–5 days before harvesting. Brucellae were removed from PIA plates using sterile PBS (sPBS) and washed twice for 10 min in sPBS.

### 2.2 Mice

Female BALB/c mice (6–8 wks of age) were purchased from Charles River Laboratory (Frederick, MD, USA) or Jackson Laboratory (Bar Harbor, ME). Breeder pairs of µMT mice on a BALB/c background were obtained from Jackson Laboratory and bred in-house. Only female mice were used for these studies since abortion can only occur with this gender. All studies using wt BM 16M were conducted under biosafety level 3 (BSL-3) containment. Mice were maintained in individually ventilated cages under HEPA-filtered-barrier conditions of 12 h of light and 12 h of darkness and were provided with food and water *ad libitum*. Upon termination of the study, all mice were humanely euthanized by metered 30% to 70% CO_2_ chamber volume/minute followed by cervical dislocation. All animal care and procedures were in accordance with institutional policies for animal health and well-being approved by the University of Florida and University of Wyoming Institutional Animal Care and Use Committees.

### 2.3 Immunofluorescence microscopy

Splenic B lymphocytes from naïve BALB/c mice were isolated to >90% purity using an EasySep^™^ Mouse Pan-B Cell Isolation Kit (Stemcell Technologies, Vancouver, CA) by following the manufacturer instructions. The purified B cells (5 x 10^5^ cells/ml) were then infected with znBM-mC at a ratio of 10:1 of bacteria to B cell in 24-well flat-bottom cell culture plate in the presence of a complete medium (CM) lacking antibiotics: RPMI 1640 (Caisson Labs, Inc, Smithfield, UT), 10 mM HEPES buffer (Caisson Labs), 10 mM nonessential amino acids (Caisson Labs), and 10 mM sodium pyruvate (Caisson Labs), and 10% fetal bovine serum (Atlanta Biologicals, Norcross, GA), and cultured for 3 hrs at 37 °C and 5% CO_2_. Following incubation, infected B cells were collected, washed in rinse buffer (Dulbeccos PBS [ThermoFisher] + 0.025% Tween 20 containing 10% goat serum and 2.5% mouse serum [Jackson ImmunoResearch Labs, West Grove, PA]), and stained with FITC-anti-mouse CD19 (clone 1D3, BioLegend, San Diego, CA) diluted in rinse buffer for 30 min at room temperature. Cells were washed in rinse buffer and coverslipped with Prolong Gold antifade reagent with DAPI (ThermoFisher). Images were captured on Keyence BZ-X810 automated fluorescent microscope (Itasca, IL).

### 2.4 Vaccination and challenge studies

Previous studies have determined that an oral prime, intranasal (IN) boost with znBM-mC was optimal for protection against virulent *Brucella* challenge (31); however, a single IN vaccination also proved effective (32). Consequently, both vaccination regimens were used for these studies. For vaccination, typically groups of 5–8 mice were used. For two-dose vaccination, mice were first orally gavaged on day 0 with 200 μL 50% saturated sodium bicarbonate solution using a 20-gauge oral gavage needle. After 10 minutes mice were then dosed with sPBS or with 1x10^9^ CFUs znBM-mC diluted to required concentration in 200 μL of volume using the same technique. For all mice receiving an IN dose, these were first anesthetized with isoflurane. For BALB/c and µMT mice given an IN boost on day 28 for two-dosed group, for single IN vaccination (5-7/group), or those used for tissue colonization studies (n = 3–6 mice/day), mice were given 5 x 10^8^ - 1x10^9^ CFU dose of znBM-mC. The bacteria were prepared to the required concentration in a volume of 30 μL, and administered dropwise into the nostrils with a micropipette. For twice-dosed mice for immunogenicity studies, mice were humanely terminated on day 56. For determination of tissue clearance, groups of mice were terminated on days 3, 7, 14, 21, and 28 post-infection.

For the pulmonary challenge on day 42 or day 56 post-primary vaccination, wt. BM 16M was prepared at a concentration of 5 x 10^4^ CFUs in 30 μL, and administered IN in the same manner as described above. Serial dilutions on PIA plates were performed after vaccination to verify dose given. Two or four weeks after pulmonary challenge, spleens and lungs from individual mice were collected, and homogenized in sterile water using a Tissue Lyser (QIAGEN, Germantown, MD). Ten-fold serial dilutions of tissue homogenates were plated on Farrell’s media (Oxoid Ltd., Basingstoke, UK) to measure extent of wt BM 16M colonization. Splenic inflammation was determined by measuring splenic weights.

### 2.5 T cell analysis

Single cell suspensions were prepared as previously described for lungs, mediastinal lymph nodes (MedLNs), and spleen (31). Briefly, tissues were placed into 2 mL tubes containing 1 mL incomplete media (ICM): RPMI 1640 (Caisson Labs, Inc, Smithfield, UT); 10 mM HEPES buffer (Caisson Labs), and 10 mM penicillin/streptomycin (Caisson Labs), and a single stainless steel bead. Tissues were homogenized (Tissue Lyser; QIAGEN), then filtered through 70 μm disposable cell strainers (Fisherbrand), and washed with ICM at 1500 RPM for 5 mins at 4 °C. Splenic red blood cells were lysed with 5 mL ammonium-chloride-potassium (ACK) buffer for 5 mins. Lung homogenates in 2 mL ICM were digested with 20 μg of Liberase TL research-grade (Roche Life Science, Indianapolis, IN) and 50 units of RNase-free DNase I (Promega Corp., Madison WI), incubated at 37 °C under 5% CO_2_, with gentle shaking for 45 mins. Lung homogenates were then filtered through 70 μm disposable cell strainers and washed with ICM. After the final wash with ICM, lung and splenic lymphocytes were resuspended in CM with 10 mM penicillin/streptomycin plus 10% fetal bovine serum.

Splenic, MedLNs, and lung lymphocytes were cultured overnight at 37 °C and 5% CO_2_ at a concentration of 5 x 10^6^ cells/mL for splenocytes and 2-2.5 x 10^6^ cells/mL for lung lymphocytes in 48-well Corning Costar plates with stimulation as follows; 10^9^ CFUs/mL heat-killed RB51 (HKRB51) followed by 4–6 hours of 2.5 ng/mL phorbol myristate acetate (PMA) (SIGMA-ALDRICH) and 250 ng/mL ionomycin (SIGMA-ALDRICH). Brefeldin A (10 μg/mL; eBioscience, San Diego, CA) was also added during the 4–6 hr stimulation to block cytokine secretion. Stimulated cells were then stained with fluorescently labeled monoclonal antibodies (mAbs) to T cell surface antigens and cytokines.

To measure antigen-presenting cell (APC) capabilities of B cells, groups of BALB/c mice (n=5/group for cell proliferation assay and n=8/group for IFN-γ release assay) were IN vaccinated with 10^9^ CFUs znBM-mC or PBS for 10 days. Lung and splenic lymphocytes were isolated as described above, and lung and splenic B cells were isolated by magnetic bead sort as described above. For B cells used as APCs, purified B cells (2.5x10^6^/ml) in CM without antibiotics were infected at 10:1 znBM-mC to B cell ratio for 3 hrs at 37 °C and 5% CO_2_. Following two washes in ICM without antibiotics, B cells were treated with 50 µg/ml gentamicin (Thermo Fisher Scientific) for 30 minutes at 37 °C and 5% CO_2_. After two washes with ICM without antibiotics, infected B cells in CM without antibiotics were treated with 50 µg/ml mitomycin C (Sigma-Aldrich) for 2 hrs. Following three washes in ICM, infected B cells were resuspended in CM. Lung and splenic CD8^+^ T cells were isolated from naïve or 10 day znBM-mC-vaccinated BALB/c mice using EasySep Mouse CD8+ T cell isolation kit (Stemcell Technologies). Pooled lung and splenic CD8^+^ T cells (200,000/well) were co-cultured with 2x10^6^ mitomycin C-treated naïve or immune B cells (1:2 ratio) in 200 µl CM with 10 U/ml of human IL-2 (PeproTech, Inc., Cranbury, NJ) in 96-well culture dishes (Corning, Inc., Corning, NY) for 3 days for proliferation assay, and 5 days (no IL-2 added) for IFN-γ release assay at 37 °C and 5% CO_2_. For the proliferation assay, cultures were pulsed with ^3^H-thymidine (0.5 μCi/well; New England Nuclear Corp., Boston, MA) for 16 hrs during the last 24 hrs of culture, and the incorporated ^3^H-thymidine was measured by scintillation counting. Data are expressed as a proliferation index of CPM of treated group divided by CPM from uninfected B cells. For the cytokine release assay, IFN-γ production was measured by ELISA as previously described (30, 31).

### 2.6 Flow cytometry analysis

To measure B cell recruitment, groups of mice (5-7/group) were IN-dosed with 5x10^4^ CFUs wt BM 16M, 10^9^ CFUs of znBM-mC, or sPBS, and terminated on day 5. Isolated lymphocytes were gated on TCRβ^-^ CD19^+^ B cells, and analyzed by flow cytometry.

Resident T cells were distinguished from circulating lymphocytes in the lungs by injection of 10 µg PerCP-coupled anti CD45 mAb (clone YW62.3, eBioscience) via the retro-orbital route. Mice were euthanized 10 mins after *in vivo* staining, and spleens and lungs were processed as described above to obtain single cell suspensions of lymphocytes. After isolation, lymphocytes were stained ex vivo with a different, noncompeting mAb clone of fluorochrome-conjugated anti-CD45 (clone 30-F11) in addition to other fluorochrome-conjugated anti-T cell mAbs. Stained cells were then subjected to flow cytometry analysis.

To identify T cell subsets induced by *Brucella* strains used for vaccination and challenge, restimulated lymphocytes were washed with FACs buffer (sPBS with 2% FBS) and then labeled with mAbs specific for TCRβ, CD45, CD4, CD8a, CD19, CD44, CD62L, CD69, CD80, CD86, CD103, MHC class I, and MHC class II for 20 mins on ice. After washing in FACs buffer, cells were then fixed with IC Fixation Buffer (eBioscience, San Diego USA), permeabilized with 1X Permeabilization Buffer (eBioscience, San Diego USA), and stained for intracellular cytokines using mAbs specific for IFN-γ and TNF-α. Fluorescence was acquired using a BD Fortessa flow cytometer with BD FACSDiva software or a Cytek Northern Lights flow cytometer using SpectroFlo software (Fremont, CA). All the fluorescently conjugated mAbs were purchased from Biolegend (San Diego USA) or eBioscience (San Diego, USA). Analysis was done using FlowJo software (Waters Biosciences, Ashland, OR, USA).

### 2.7 Adoptive transfer of B cells

To study the relevance of B cells adoptively transferred into µMT mice, two studies were performed. In the first study, analyses were done on days 14 and 42 post-primary znBMZ vaccination. Groups of mice (n = 5/group) included PBS only, znBMZ only, and adoptive transfer of normal BALB/c B cells given by the retro-orbital (RO) route on day -1 and by the IN route on day 28. Groups were orally vaccinated on day 0 with 10^9^ CFUs znBMZ. Three groups of µMT mice (PBS only; znBMZ-vaccinated only; and B cell recipients znBMZ-vaccinated) were evaluated on day 14 by flow cytometry for memory CD4^+^ and CD8^+^ T cell subsets. Three other groups of orally znBMZ-vaccinated µMT mice were IN boosted with znBMZ on day 29. One group of B cell µMT recipients were given a second adoptive transfer of naïve BALB/c B cells on day 28 by the IN route, and an additional of orally znBMZ-vaccinated µMT mice was given a single adoptive transfer of naïve BALB/c B cells also on day 28. All of these remaining groups were then evaluated by flow cytometry for memory CD4^+^ and CD8^+^ T cell subsets on day 42.

In the second experiment to measure donor B cells’ contribution to protection against challenge, control groups of BALB/c and µMT mice (5-8/group) were given a single IN vaccination with sPBS or 10^9^ CFUs znBM-mC as described above. Vaccinated recipient µMT mice were adoptively transferred with either naïve B cells or with immune B cells, which were derived from single cell suspensions of lung and splenic lymphocytes prepared, respectively from naïve or znBM-mC-vaccinated BALB/c mice. Lung and splenic B cells were isolated using magnetic bead negative selection (EasySep^™^ Mouse Pan-B Cell Isolation Kit, Stemcell Technologies). An aliquot of cells was stained for purity by flow cytometry, and confirmed the B cell purity > 90%. The purified B cells were resuspended in sPBS at a concentration of 2-6x10^6^ cells/30 µL; all µMT recipients received the same number of B cells. At six wks post-vaccination, recipient naïve or znBM-mC-vaccinated BALB/c and µMT mice received a single IN administration of purified B cells while under isoflurane anesthesia. Twenty-four hours post-adoptive transfer, mice were challenged via the IN route with 5 x 10^4^ CFUs BM 16M in 30 µl sPBS. Lungs and spleens were collected two weeks later, and processed for tissue CFUs and T cell analysis as previously described.

### 2.8 Statistical analysis

Distribution of data was analyzed for normal distribution using various tests (Anderson-Darling, Shapiro-Wilk, Kolmogorov-Smirnov). For normally distributed data One-way analysis of variance (ANOVA) with multiple comparison tests were used to compare groups depending on the data sets using Tukey’s multiple comparisons test or Holm-Sidak method for pairwise comparisons. For data that did not follow a normal distribution, Kruskall-Wallis test was used to analyze differences in medians followed by multiple comparison tests. GraphPad Prism and SigmaPlot 15 were used for statistical analysis and to generate graphs.

## 3 Results

### 3.1 IN znBM-mC is readily cleared from lungs and spleen within four wks of dosing

Previous studies have established that B cell deficient (µMT) mice are more resistant to infection against wt *Brucella* (39, 42). As these studies generally focused on intraperitoneal infection, the investigation described here examines infectivity following mucosal application, i.e., by the IN and oral routes. Additionally, we queried whether the attenuated, double mutant strain, znBM-mC, alters the infection pattern in both μMT and BALB/c mice. Groups of μMT and BALB/c mice were IN dosed with 1 x 10^9^ CFUs znBM-mC. The extent of tissue colonization was measured at varying days post-infection out to 28 days. No differences in lung and spleen colonization by znBM-mC between both mouse strains were observed (**Figures 1A, B**). Thus, znBM-mC is cleared within 28 days of IN infection.

**Figure 1:**
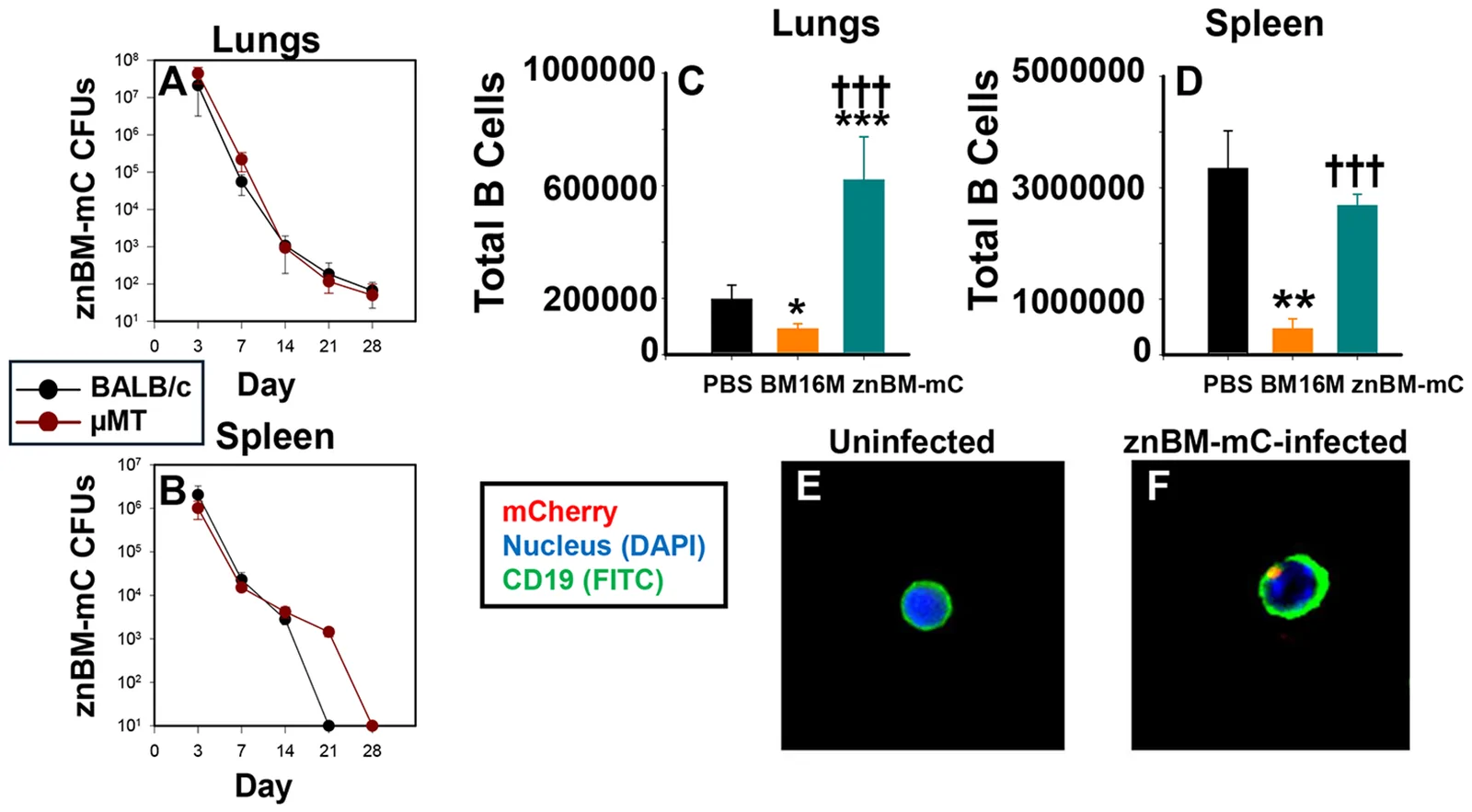
The double-mutant *B. melitensis strain*, znBM-mC readily clears from wild-type and µMT mice, and promotes B cell recruitment to the lungs. Colonization by znBM-mC following a single nasal infection of BALB/c and B cell-deficient (µMT) **(A)** lungs and **(B)** spleen significantly declines over time; n = 3–5 mice/species/time point (days 3, 7, 14, 21, and 28), P < 0.05. B cells from **(C)** lungs and **(D)** spleen from BALB/c mice (5-8/group) infected IN with 5x10^4^ CFUs wt BM 16M or 10^9^ CFUs znBM-mC are depicted. B cells from wt BM 16M-infected mice are reduced compared to naive mice unlike those from znBM-mC-dosed mice. A representative of 4 experiments is shown; ***P < 0.001, **P = 0.012, *P < 0.05 vs PBS-dosed mice; ^†††^P < 0.001 vs. wt BM 16M-infected mice. **(E, F)** Immunofluorescent microscopy (500x) of CD19^+^ B cells are **(F)** susceptible to infection with znBM-mC (mCherry), CD19 (FITC), and nuclear DAPI stain (blue).

### 3.2 IN znBM-mC vaccination results in B cells infiltrating the lungs

To understand how the host responds to znBM-mC, a study was performed to measure the early lymphocyte response. Five days following IN vaccination with znBM-mC, a significant influx of B lymphocytes was observed in the lungs (P < 0.001), but not in mice infected with wt BM 16M (**Figure 1C**). In fact, lung B cells in BM 16M-infected mice were significantly reduced relative to naïve lung B cell levels (P < 0.05). The same held true for the spleen, where B cell levels from wt BM 16M-infected mice were reduced by 7-fold (P = 0.012) relative to levels in PBS-dosed mice (**Figure 1D**). Splenic B cell levels did not significantly change for znBM-mC-vaccinated mice.

### 3.3 B Cells are susceptible to infection by znBM-mC

Others have shown that wt *Brucella* can infect B cells (39, 40). To ascertain if B cells are susceptible to infection by znBM-mC, naïve splenic B cells were isolated and infected with znBM-mC at 10:1 infection ratio. Immunofluorescence microscopy revealed that znBM-mC can infect naïve CD19^+^ B cells (**Figure 1F**).

### 3.4 Vaccination of μMT mice with znBM-mC fails to protect against wt BM 16M challenge

To investigate the possible role of B cells in protection against virulent wt BM 16M challenge, groups of BALB/c and µMT mice were vaccinated by our standard method (30, 31), oral prime followed by IN boost or alternatively, a separate group was given a single IN dose similar to that previously described (32; **Figure 2A**). On day 42 for single-dosed mice or day 56 for two-dosed mice post-primary vaccination, all mice were subjected to pulmonary challenge with wt BM 16M (**Figure 2A**), and 2–4 wks later, lungs and spleens were collected from individual mice (**Figures 2B–G**). Twice znBM-mC-vaccinated BALB/c mice showed a 2–3 log reduction in tissue colonization by wt BM 16M (**Figures 2B, C**). In contrast, similarly znBM-mC-vaccinated µMT mice showed no significant difference in tissue colonization relative to PBS-dosed µMT mice both in the lungs or spleen. Likewise, single-dosed BALB/c mice showed reduced lung and splenic colonization evident by 2–3 log reduction in wt BM 16M colonization (**Figures 2E, F**). When similar vaccination regimen was applied to µMT mice, znBM-mC failed to confer protection, and tissue colonization did not significantly differ in either the lungs or spleens (**Figures 2E, F**). Infection with wt BM 16M induced a significant inflammatory response in PBS-dosed BALB/c mice, evident by their increased splenic weights, but these weights were reduced by 45% in znBM-mC-vaccinated BALB/c mice (**Figures 2D, G**). However, znBM-mC-vaccinated μMT mice challenged with wt BM 16M showed no significant increase in their splenic weights when compared with PBS-dosed controls. Given the absence of B cells from their spleens, µMT have inherently reduced splenic weights relative to BALB/c mice. Thus, these data show that znBM-mC vaccination is unable to confer protective immunity in µMT mice.

**Figure 2:**
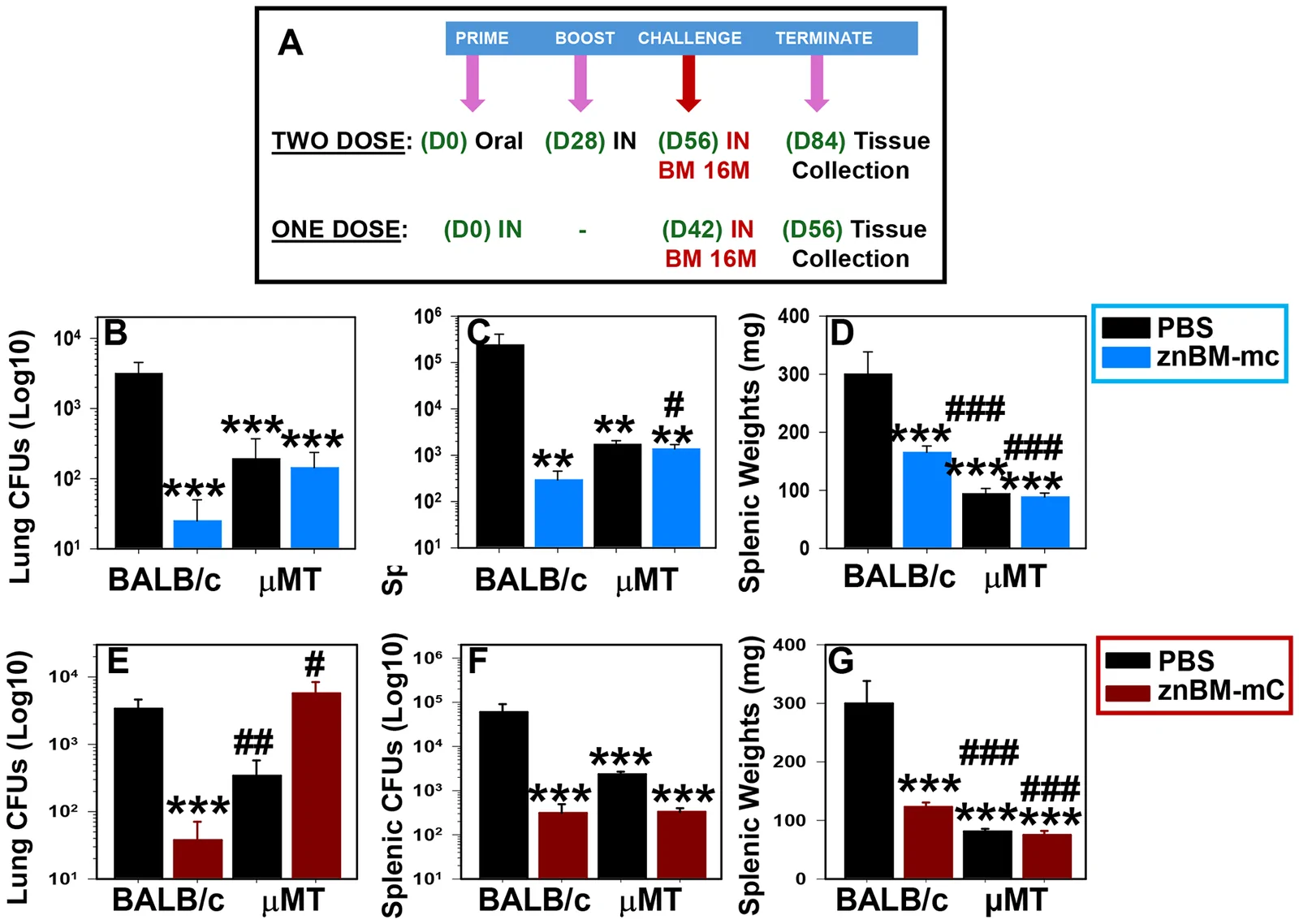
Vaccination with znBM-mC fails to protect µMT mice against wt BM 16M challenge, and is only efficacious in BALB/c mice. **(A–D)** BALB/c and µMT mice on a BALB\c background (5–8 mice/group) were orally primed on day 0 with 10^9^ CFUs of znBM-mC and IN boosted on day 28 with 5 x 10^8^ CFUs. **(A, E–G)** Separate groups of BALB/c and µMT mice (5-8/group) were given PBS or a single IN dose of 10^9^ CFUs znBM-mC on day 0. All mice were subjected to pulmonary challenge with 5 x 10^4^ CFUs of wt *B. melitensis* 16M on day 56 **(B–D)** or on day 42 for single IN dosed mice **(E–G)**. Lungs and spleens were collected for CFU enumeration on day 84 for twice-vaccinated mice **(B–D)** or on day 56 for single dosed mice **(E–G)**. Bacterial loads in both **(B, E)** lungs and **(C, F)** spleens, and **(D, G)** splenic weights were measured. Data are representative of two independent experiments. ANOVA with one-way ANOVA Tukey’s multiple comparisons. test was performed. Error bars represent standard error of the mean (SEM): ***P < 0.001, **P < 0.01 vs. PBS-dosed BALB/c mice; ^###^P < 0.001, ^##^P < 0.020, ^#^P < 0.05 vs. znBM-mC-vaccinated BALB/c mice.

### 3.5 Lung T cell subsets do not differ in μMT from BALB/c mice after vaccination with znBM-mC

Cell-mediated immunity is critical for clearance of *Brucella* infections (1, 2, 5). To determine whether the absence of B cells compromises vaccine-induced T cell immunity, T cell responses by μMT and BALB/c mice were examined post-vaccination with znBM-mC. Groups of μMT and BALB/c mice were IN vaccinated once (day 0) or twice (days 0 and 28), and on days 14 and 42, splenic and lung lymphocytes were isolated, stained, and analyzed by flow cytometry to characterize the T cell response (**Figures 3A, B**). Examination of the total numbers of CD4^+^ T cells on day 42 revealed that vaccinated BALB/c mice showed a significant reduction relative to unvaccinated mice (**Figure 3C**). In contrast, a significant influx of CD4^+^ T cells into the µMT lungs was observed on day 14 relative to day 0, and these were reduced at day 42 (**Figure 3C**). For BALB/c CD8^+^ T cells, no significant differences in total numbers were observed among the three timepoints examined (**Figure 3D**). However, in μMT lungs, the total number of CD8^+^ T cells increased after the primary vaccination, and was reduced following the booster vaccination, but still relatively greater than at pre-vaccination (**Figure 3D**).

**Figure 3:**
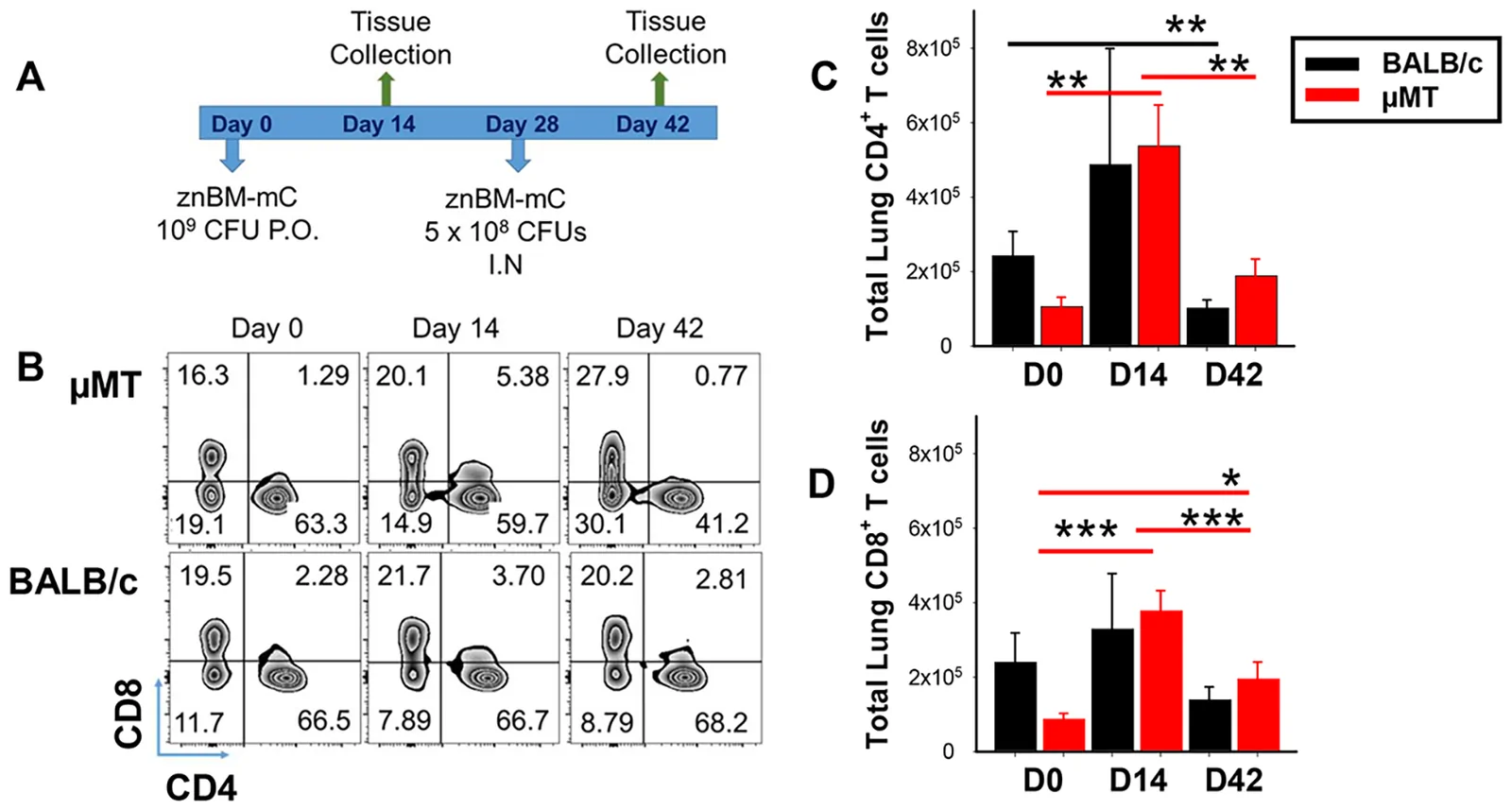
The expansion of lung T cells during early and late time points is unaffected by the absence of B cells. **(A)** Groups of BALB/c and μMT mice (5/time point) were orally primed on day 0 with 10^9^ CFUs of znBM-mC, and IN boosted on day 28 with 5x10^8^ CFUs znBM-mC. Lungs were collected and lymphocytes isolated for FACS analysis at the indicated timepoints. **(B)** FACS plots representing lung CD4^+^ and CD8^+^ T cells on days 0, 14, and 42 are shown. Total BALB/c and μMT **(C)** CD4^+^ and **(D)** CD8^+^ T cells on days **(D)** 0, 14, and 42 post-primary vaccination are depicted. ANOVA with one-way ANOVA Tukey’s multiple comparisons test was performed; ***P ≤ 0.001, **P ≤ 0.003, *P < 0.050 between the indicated groups.

### 3.6 znBM-mC-infected B cells act as APCs

To assess whether znBM-mC-infected B cells can serve as APCs, flow cytometry analysis was first performed on lung B cells isolated from znBM-mC-infected mice to determine if these become activated. Five days after IN vaccination, the MFI for co-stimulatory molecule, CD80, was significantly increased by mC^+^ B cells compared to mC^-^ B cells (**Figure 4A**), while no differences in MFIs for CD86 were observed. Increases in MFIs for MHCI, MHCII, and CD11b were also found by lung mC^+^ B cells (**Figure 4A**). With the increased activation status of B cells, we queried whether CD8^+^ T cell proliferation is impacted. Groups of mice were IN vaccinated with znBM-mC or PBS for 10 days, and CD8^+^ T cells were purified from lungs and spleens. CD19^+^ B cells were purified from naïve mice, and these served as an infected APC source. A fourfold increase in proliferation by pooled CD8^+^ T cells was observed when co-cultured with znBM-mC-infected B cells (**Figure 4B**). In contrast, naïve CD8^+^ T cells minimally proliferated in response to znBM-mC-infected B cells. To further measure APC function, an IFN-γ release assay was performed using pooled lung and splenic CD8^+^ T cells from 10-day vaccinated mice. B cells from naïve or znBM-mC-infected mice were tested for their ability to stimulate IFN-γ when B cells were used as APCs for immune CD8^+^ T cells (**Figure 4C**). Following 5 days of *in vitro* culture using znBM-mC-infected immune B cells as APCs, the greatest IFN-γ production by CD8^+^ T cells was obtained compared to CD8^+^ T cells co-cultured with infected naïve B cells (**Figure 4C**). Minimal IFN-γ production was obtained when CD8^+^ T cells were co-cultured with uninfected immune or naïve B cells. No IFN-γ was detected by uninfected B cells cultured alone. Thus, these results show that B cells can serve as APCs for immune CD8^+^ T cells.

**Figure 4:**
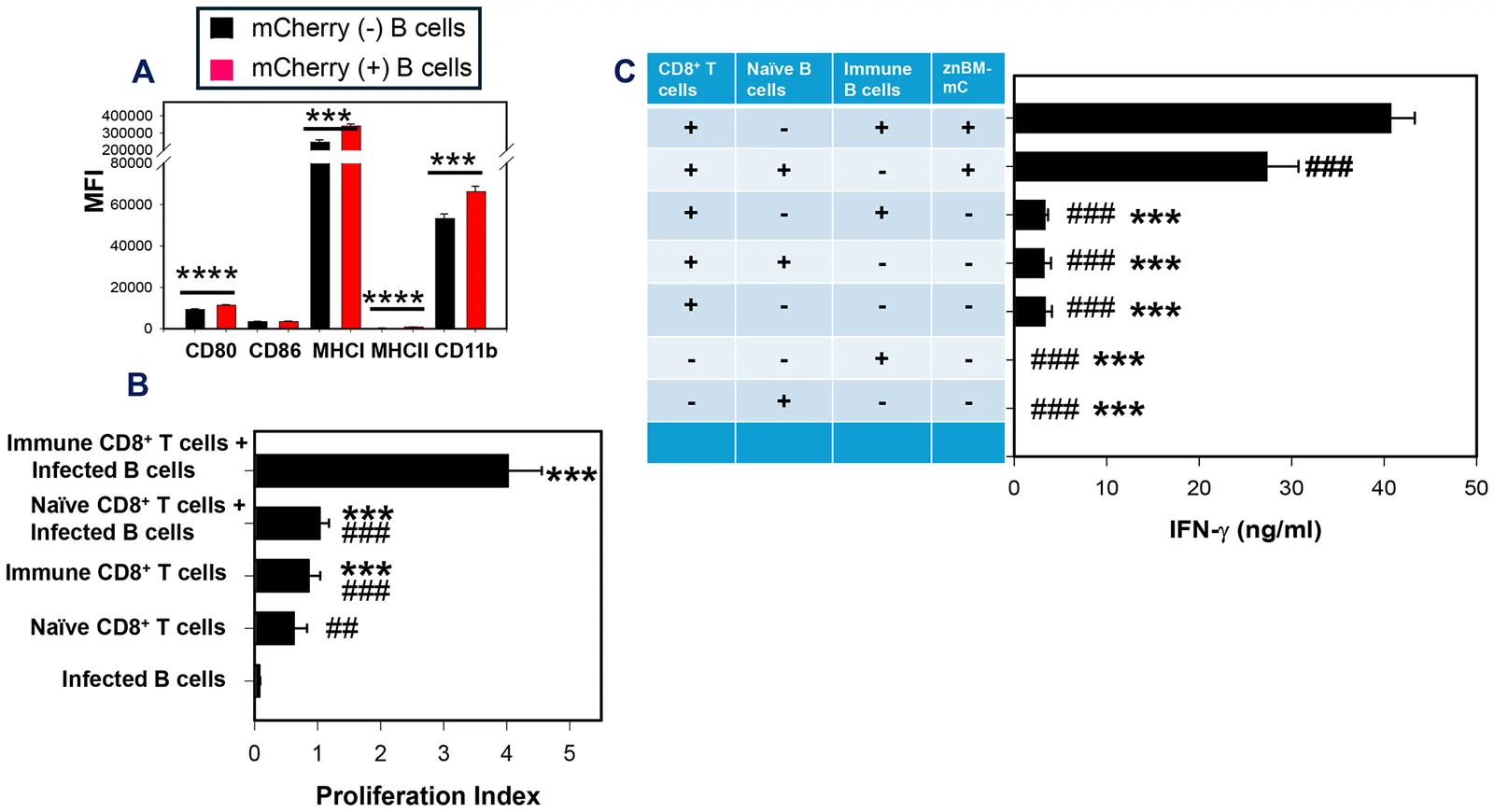
znBM-mC-infected B cells act as antigen-presenting cells (APCs). **(A)** BALB/c mice (n=5) were IN znBM-mC-infected, and 5 days later, mCherry^-^ and mCherry^+^ lung B cells were measured by flow cytometry for extent of activation (MFIs) for CD80, CD86, MHCI, MHCII, and CD11b. A one-way ANOVA model and Holm-Sidak method were used for statistical analysis: ****P < 0.0001, ***P ≤ 0.005 versus mCherry^+^ B cells. **(B)** Groups of BALB/c mice (n=5/group) were IN dosed with PBS or IN vaccinated with znBM-mC, and 10 days later, lung and splenic CD8^+^ T cells were purified from each group. Purified naïve B cells were infected with znBM-mC for 3 hrs,and gentamicin-treated to kill extracellular bacteria. CD8^+^ T cells were co-cultured with mitomycin C-treated infected B cells at a 1:2 T:B cell ratio for 3 days and pulsed with ^3^H-thymidine for the last 16 hrs. Data are expressed as a proliferation index: CPM from treated cultures were divided by CPM derived from uninfected B cells. A one-way ANOVA model and Holm-Sidak method were used for statistical analysis: ***P < 0.001 versus infected naive B cells; ^###^P < 0.001, ^##^P = 0.005 versus immune CD8^+^ T cells co-cultured with infected B cells. **(C)** Additional groups of BALB/c mice (n=8/group) were IN vaccinated with znBM-mC as described in **(B)**, and CD8^+^ T cells were subsequently purified from lungs and spleens. Naïve and immune B cells purified as previously described, then infected with znBM-mC, and mitomycin B-treated as described in **(B)**. CD8^+^ T cells were co-cultured with mitomycin C-treated infected B cells (from naïve or vaccinated mice) at a 1:2 T:B cell ratio for 5 days, and cell culture supernatants were measured for IFN-γ by ELISA. Control groups included CD8^+^ T cells co-cultured with infected naive B cells or uninfected naïve or immune B cells, and CD8^+^ T cells, naïve B cells, or immune B cells cultured alone. A one-way ANOVA model and Holm-Sidak method were used for statistical analysis: ^###^P < 0.001 versus immune CD8^+^ T cells co-cultured with infected immune B cells; ***P < 0.001 versus immune CD8^+^ T cells co-cultured with infected naive B cells.

### 3.7 µMT mice exhibited greater proinflammatory T cells

Examination of their cytokine profiles following znBM-mC vaccination revealed that both groups of vaccinated mice stimulated the induction of lung IFN-γ^+^ and TNF-α^+^ CD4^+^ and CD8^+^ T cells (**Figures 5A–D**). Interestingly, znBM-mC-vaccinated µMT mice showed significantly greater lung IFN-γ^+^ CD8^+^, TNF-α^+^ CD4^+^, and TNF-α^+^ CD8^+^ T cells when compared to znBM-mC-vaccinated BALB/c mice (**Figures 5B–D**). Additionally, both BALB/c and µMT showed induction of double-positive, IFN-γ^+^ TNF-α^+^ CD4^+^ and CD8^+^ T cells, and these were significantly greater in vaccinated µMT mice (**Figures 5E, F**). Thus, these results show that proinflammatory T cells are stimulated in znBM-mC-vaccinated µMT mice.

**Figure 5:**
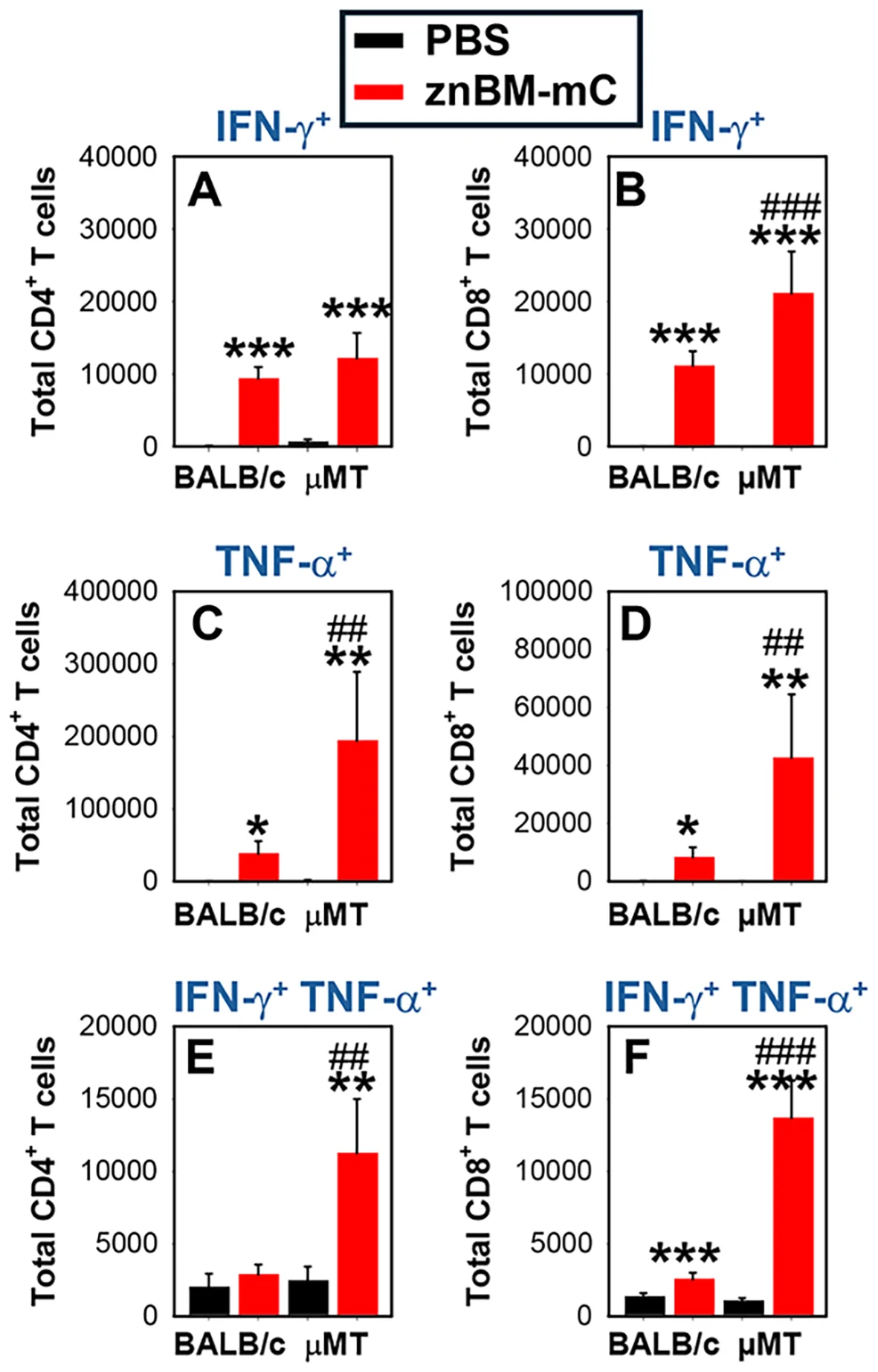
Vaccination with znBM-mC induces increased IFN-γ^+^ and TNF-α^+^ CD4^+^ and CD8^+^ T cells in the lungs. Groups of BALB/c and µMT mice (5/group) were orally primed, nasally boosted as described in **Figure 2**. On day 56 post-primary vaccination, lungs were evaluated for the presence of **(A, B)** IFN-γ^+^, **(C, D)** TNF-α^+^, **(E, F)** IFN-γ^+^ TNF-α^+^ **(A, C, E)** CD4^+^ and **(B, D, F)** CD8^+^ T cells. A one-way ANOVA model was used for statistical analysis: ***P < 0.001, **P < 0.005, *P < 0.05 vs. PBS-dosed mice within the same mouse strain. A combination of two experiments is presented, and analyzed by one-way ANOVA Tukey’s multiple comparisons test was performed; ^###^P < 0.001, ^##^P < 0.005 vs. znBM-mC-vaccinated BALB/c mice.

### 3.8 Vaccination of µMT mice with znBM-mC induces lung memory T cells

Groups of BALB/c and µMT were vaccinated using the two-dose regimen (**Figure 6A**). To determine resident memory T cells, on day 56, mice were given an IV fluorochrome-labeled anti-CD45 mAb ten minutes prior termination. Lung lymphocytes were gated on CD45^-^ TCRβ^+^ CD19^-^ T cells (**Figures 6B, C**) for the induction of memory phenotypes: CD44^hi^ CD62L^lo^ CD69^-^ effector memory T cells (TEMs; **Figures 5D, E**); CD44^hi^ CD62L^lo^ CD69^+^ resident memory T cells (TRMs; **Figures 6F, G**); and CD44^hi^ CD62L^+^ central memory T cells (TCMs; **Figures 6H, I**). TRMs, TCMs, and CD8^+^ TEMs were induced in both mouse strains to equivalent levels. Only CD4^+^ TEMs were significantly reduced in vaccinated µMT mice (**Figure 6D**).

**Figure 6:**
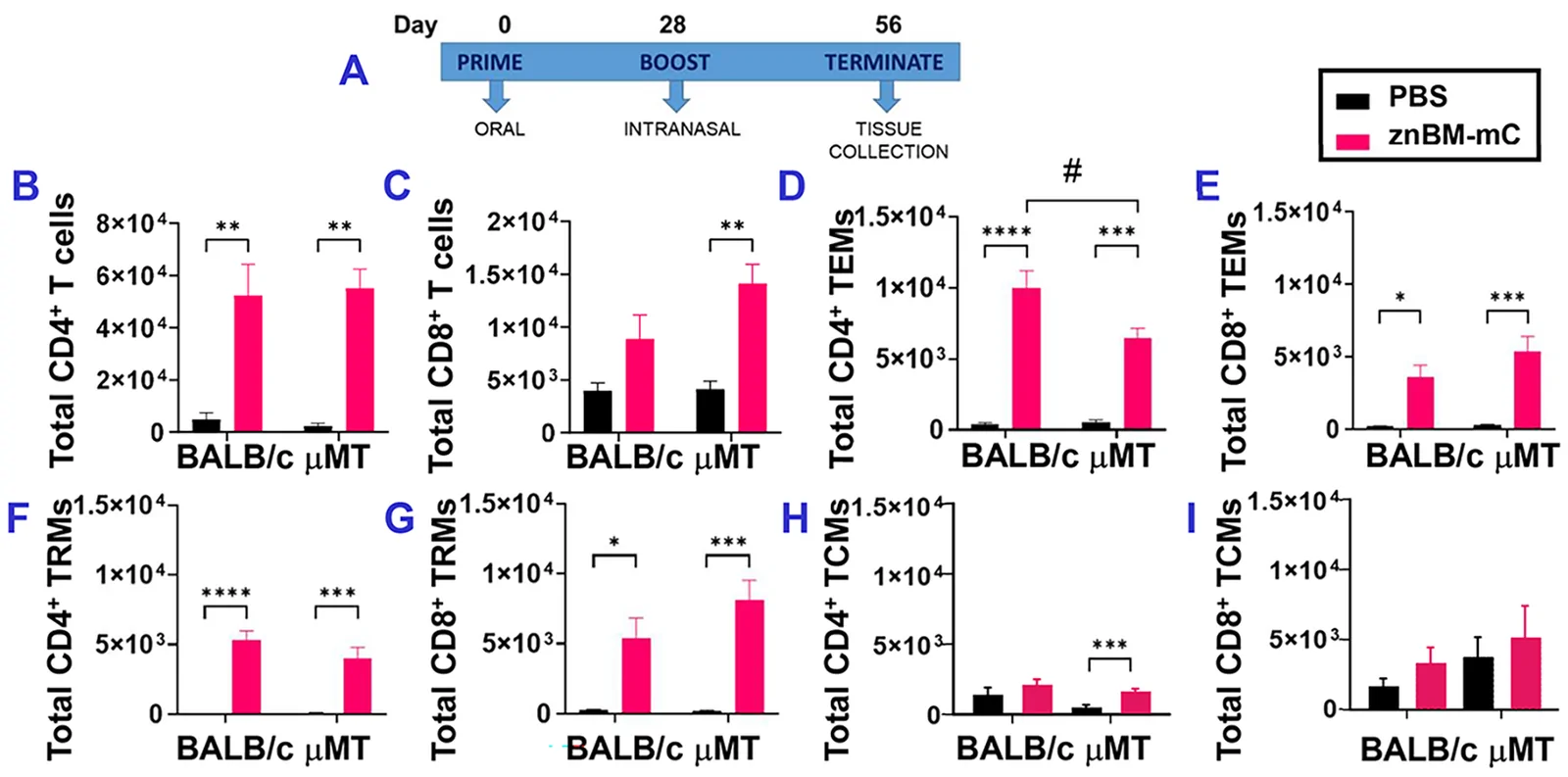
The lack of B cells does not compromise lung T cell memory differentiation by znBM-mC-vaccinated mice. **(A)** Groups of BALB/c and μMT mice (5 mice/group) were vaccinated as described in **Figure 2A**. The study was terminated on day 56, and lung lymphocytes were collected for FACS analysis. **(B)** Total lung CD4^+^ and **(C)** CD8^+^ T cells from PBS-dosed or znBM-mC-vaccinated BALB/c and μMT mice were enumerated. BALB/c and μMT lung lymphocytes were subsequently analyzed for the presence of CD44^hi^ CD62L^lo^ CD69^-^ effector memory T cells (TEMs): total lung **(D)** CD4^+^ and **(E)** CD8^+^ TEMs. CD44^hi^ CD26L^lo^ CD69^+^ resident memory T cells (TRMs) were measured: total lung **(F)** CD4^+^ and **(G)** CD8^+^ TRMs. CD44^hi^ CD62L^+^ central memory T cells (TCMs) were measured: total lung **(H)** CD4^+^ and **(I)** CD8^+^ TCMs. A one-way ANOVA model and Holm-Sidak method were used for statistical analysis: ****P < 0.0001, ***P < 0.0005, **P < 0.005, *P < 0.05 vs. PBS-dosed mice within the same mouse strain; ^#^P < 0.05 between vaccinated mouse strains.

### 3.9 Adoptive transfer of naïve B cells into μMT mice enhances CD4^+^ and CD8^+^ T cells in the mediastinal LNs following vaccination

Groups of µMT mice were dosed using the oral prime, nasal boost strategy for znBMZ vaccination (**Supplementary Figures 1A, B**). On day -1, some µMT mouse recipients were adoptively transferred with naive B cells via the RO route (Groups 3 and 5), and a second transfer performed on day 28 (one day prior to boost) via the nasal route (Group 5). A separate group of mice received naive B cells only on day 28 via the nasal route prior to booster vaccination (Group 6). PBS and vaccinated control mice (Groups 1, 2, and 4) did not receive any adoptive transfers. Groups 1–3 mice were orally vaccinated on day 0 and terminated on day 14. Groups 4–6 received an IN znBMZ boost on day 29. T cells from the lungs, MedLNs, and spleens were examined on days 14 and 42 for memory T cell phenotype. No obvious changes in the percentage of CD4^+^ and CD8^+^ T cells were observed in the lungs as a consequence of vaccination and/or B cell adoptive transfer, but notable changes were observed for MedLN CD8^+^ T cells (**Supplementary Figures 1I–N**). For single-dosed µMT recipients on day 14, significant increases in percentages of MedLN CD4^+^ and CD8^+^ T cells were observed relative to the PBS-dosed µMT mice (**Supplementary Figures 1I, L**); no difference was observed in CD4^+^ or CD8^+^ T cells in vaccinated µMT (Group 2) mice. Increases in percentage of splenic CD4^+^ T cells in both vaccinated µMT mice and µMT recipients were also observed (**Supplementary Figure 1O**).

On day 42, both lung and MedLN CD4^+^ Tems were significantly increased compared to naïve controls (**Supplementary Figures 1D, J**). MedLN, not lung nor splenic, CD8^+^ T cells from vaccinated µMT mice and µMT recipients were significantly increased relative to PBS-dosed µMT mice (**Supplementary Figure 1L**). In fact, vaccinated µMT recipients given a single nasal adoptive transfer of naïve B cells on day 28 showed a significant increase in MedLN CD8^+^ T cell frequency than vaccinated µMT mice. No significant changes in MedLN and splenic CD8^+^ Tems were observed. Increases in the frequency of lung CD8^+^ Tems were found for vaccinated µMT mice and vaccinated µMT recipients given two adoptive transfers (**Supplementary Figure 1G**).

On day 42, lung CD4^+^ Tcms and MedLN CD8^+^ Tcms were increased in µMT recipients given a single nasal adoptive transfer of naïve B cells on day 28 (**Supplementary Figures 1E, N**). Splenic CD8^+^ Tcms were also increased in µMT recipients given a single nasal adoptive transfer of naïve B cells on day 28 and twice vaccinated µMT mice (**Supplementary Figure 1T**). Thus, these data show that adoptive transfer of B cells can aid in improving CD4^+^ and CD8^+^ memory T cell frequencies.

### 3.10 Reconstitution of znBM-mC-vaccinated μMT mice with BALB/c B cells into the lungs prior to challenge restores the protective immune response to BM 16M challenge

The reduction in protection observed in μMT mice cannot be attributed to a lack of T cell responses to znBM-mC as varied T cell subsets were induced to near equivalence except for CD4^+^ TEMs, which were slightly reduced. Thus, we queried whether lung B cells elicited by znBM-mC vaccination contribute to the vaccine-induced protection against pulmonary challenge with virulent wt BM 16M. To test for this, groups of BALB/c and µMT mice were subjected to a single vaccination regimen with znBM-mC similar to **Figure 2A**. On day 42, one day before challenge, groups of znBM-mC-vaccinated μMT mice were IN adoptively transferred into the lungs with either B cells derived from naïve or znBM-mC-vaccinated BALB/c mice. To discern if vaccination is required of µMT mice, a separate group of unvaccinated (PBS-dosed) µMT recipients were adoptively transferred with donor immune BALB/c B cells. On day 43, all PBS-dosed or znBM-mC-vaccinated BALB/c and µMT mice, and µMT recipients adoptively transferred with naïve or immune B cells were subjected to pulmonary challenge with wt BM 16M. Then, two weeks post-challenge (day 57), lungs and spleens were collected, and CFU enumeration was performed among the groups (**Figures 7A, B**). Consistent with results in **Figure 2E**, no significant reduction in brucellae colonization of the lungs was observed between naïve and znBM-mC-vaccinated μMT mice (**Figure 7A**). In contrast, znBM-mC-vaccinated BALB/c mice showed a protective response in both tissues. Interestingly, donor B cells from either znBM-mC-vaccinated or naïve BALB/c mice aided in the reduction of brucellae colonization of the lungs in znBM-mC-vaccinated μMT recipients to levels similar to those in znBM-mC-vaccinated BALB/c mice (**Figure 7A**). Adoptive transfer of naïve or immune B cells into znBM-mC-vaccinated μMT mice also showed significant reduction in splenic brucellae colonization from znBM-mC-vaccinated μMT mice, and vaccinated μMT recipients of immune B cells showed >300-fold reduction in splenic colonization (**Figure 7B**). When donor immune B cells were adoptively transferred into naïve µMT recipients, these had no impact on the extent of brucellae colonization of the lungs (**Figure 7A**), and only a slight impact in the spleen when compared to those in PBS-dosed µMT mice (**Figure 7B**). Thus, adoptive transfer of immune B cells into naïve µMT recipients failed to confer protection, and protection with immune B cells only occurs if mice have been previously vaccinated. Vaccination of BALB/c mice with znBM-mC did significantly reduce the extent of splenic inflammation relative to wt BM 16M challenged BALB/c mice (**Figure 7C**). In contrast, the splenic weights for znBM-mC-vaccinated µMT mice were notably increased, but the adoptive transfer of naïve B cells was able to significantly reduce splenic weights in these µMT recipients (**Figure 7C**). Thus, adoptive transfer of naïve or immune B cells proved therapeutic in reducing brucellae colonization of the lungs of vaccinated µMT mice.

**Figure 7:**
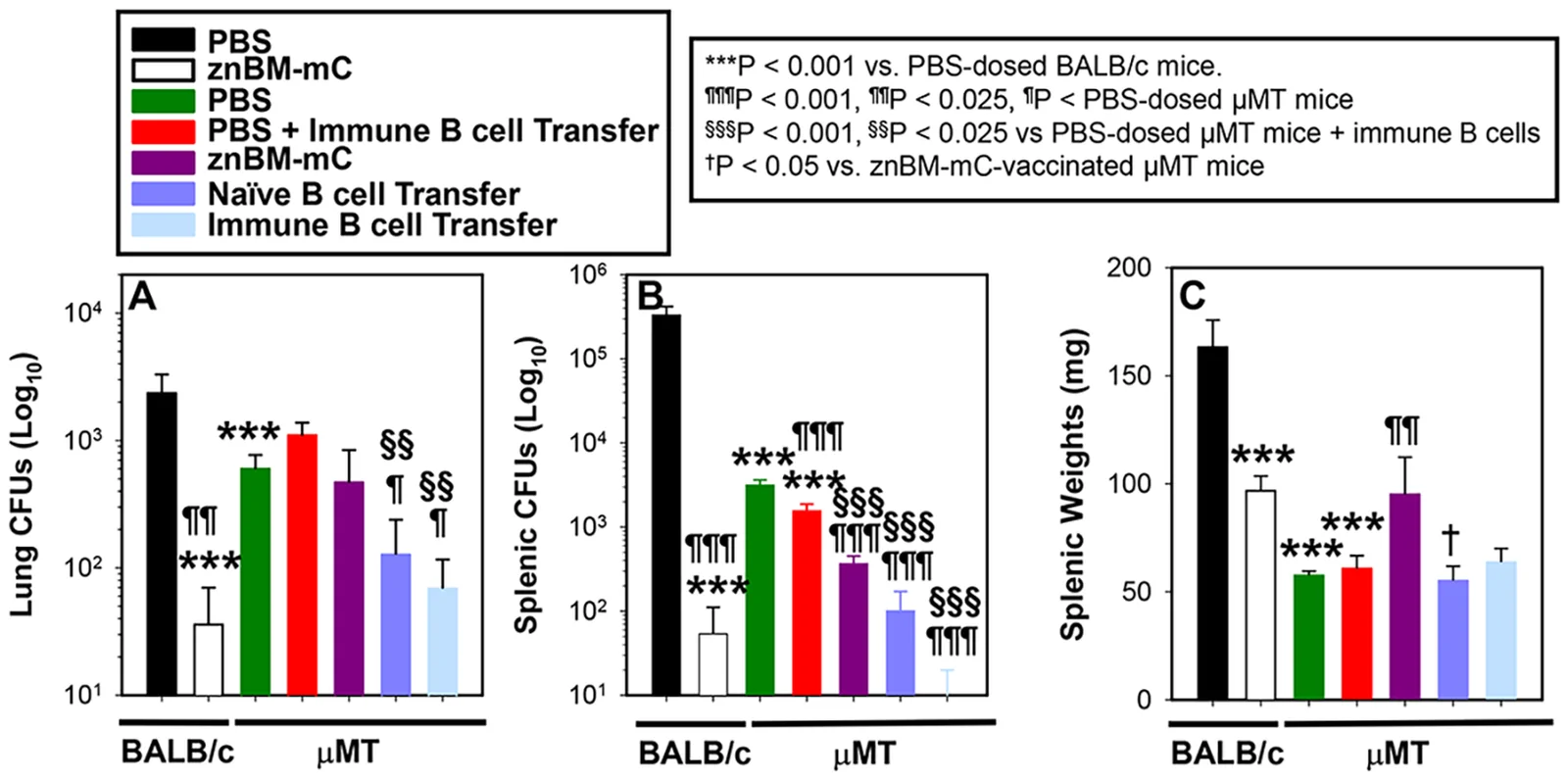
Adoptive transfer of purified B cells into znBM-mC-vaccinated µMT mice prior to pulmonary challenge with wt *B. melitensis* 16M dose results in reduced wt brucellae colonization of the lungs and spleen. BALB/c and μMT mice given a single IN vaccination with PBS or znBM-mC. An additional group of naïve µMT mice given PBS were IN recipients of immune BALB/c B cells, and two vaccinated μMT groups received IN adoptive transfer of purified B cells from naïve or znzBM-mC-vaccinated BALB/c mice one day prior to challenge with wt BM 16M. Lungs and spleens were collected 2 weeks post-challenge for CFU enumeration. **(A)** Lung and **(B)** splenic CFUs from each test group are depicted. **(C)** Splenic weights of experimental groups are shown. Data are representative of two separate experiments using 5–8 mice per group. A one-way ANOVA model and Holm-Sidak method were used for statistical analysis: ***P < 0.001 vs. PBS-dosed BALB/c mice; ^¶¶¶^P < 0.001, ^¶¶^P < 0.025, ^¶^P < 0.05 vs. PBS-dosed µMT; ^§§§^P < 0.001, ^§§^P < 0.025 vs PBS + immune B cell transfer µMT mice; and ^†^P < 0.05 vs. znBM-mC-vaccinated µMT mice.

T cells present in the lungs 2 wks post-challenge were also evaluated (**Figure 8**). In the lungs from znBM-mC-vaccinated µMT mice, a significant influx of resident memory (IV CD45^-^) CD4^+^ and CD8^+^ T cells were detected relative to PBS-dosed µMT mice (**Figures 8A, C**), yet lung colonization by wt BM 16M remained unchanged. Interestingly, the IV CD45^-^ CD8^+^ T cells were significantly increased relative to PBS-dosed µMT mice. No significant differences were evident with IV CD45^+^ CD4^+^ T cells (**Figure 8B**), but the IV CD45^+^ CD8^+^ T cells in the µMT recipients of immune B cells did show a significant increase relative to PBS-dosed BALB/c mice (**Figure 8D**). Given these findings, the µMT recipients of immune B cells did show enhanced CD8^+^ T cell numbers.

**Figure 8:**
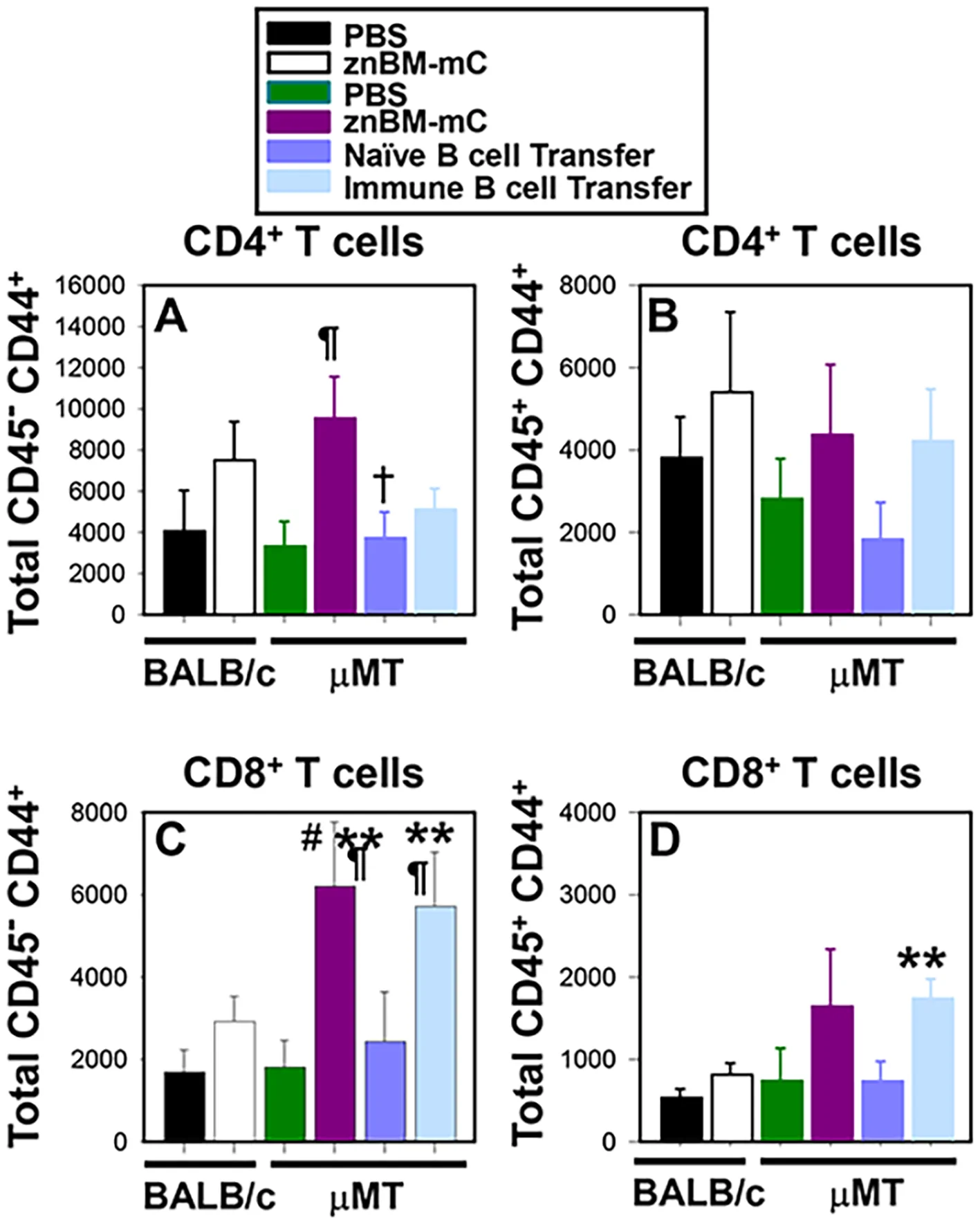
T cells present in the lungs after challenge of mice adoptively transferred with purified B cells into znBM-mC-vaccinated µMT mice. Analysis of lung T cells from **Figure 7** are described here. Lung T cells recovered two wks post-challenge revealed no significant differences in **(A)** CD45^-^ or **(B)** CD45^+^ CD44^+^ CD4^+^ T cells in µMT mice relative to znBM-mC-vaccinated BALB/c mice. **(C)** znBM-mC-vaccinated µMT mice and µMT recipients of immune B cells showed increased CD45^-^ CD44^+^ CD8^+^ T cells. **(D)** No differences in immune mice or µMT recipient in CD45^+^ CD44^+^ CD8^+^ T cells were observed. A one-way ANOVA model and Holm-Sidak method were used for statistical analysis: **P ≤ 0.015 vs. PBS-dosed BALB/c mice; ^#^P < 0.05 vs. znBM-mC-vaccinated BALB/c mice; ^¶^P < 0.05 vs PBS-dosed µMT mice; and ^†^P < 0.05 vs. znBM-mC-vaccinated µMT mice.

## 4 Discussion

While aided in elimination of disease by test and cull method in the US during the mid-twentieth century (1, 2), conventional brucellosis vaccines show only about 70% efficacy even after revaccination (2). Due to its insidious nature, brucellosis has spread into wildlife impacting the Greater Yellowstone Ecosystem (GYE), and livestock vaccines have proved ineffective in bison and elk (1, 43). Thus, livestock herds within the bordering states of the GYE have the added burden of increased susceptibility to brucellosis infection. Brucellosis can also be contracted by humans, mostly via incidental contact with infected animals, needlestick, or consumption of unpasteurized dairy products (3, 13–16, 44). Since no human vaccines exist, any improved understanding of *Brucella*’s pathogenicity and protection can enable development of vaccines for both humans and livestock.

To aid in this effort to develop improved strategies for immune protection and a universal vaccine for brucellosis, we considered two essential traits. First was the development of suitable mutations that could sustain a live vaccine presence for sufficient duration to elicit a potent cell-mediated immune response to deter *Brucella*’s natural resistance mechanisms (1, 18). Second, was testing a mucosal route of vaccination since brucellosis is contracted mostly from a mucosal exposure (1, 12, 45). Conventional mode of vaccination and experimental testing has relied mostly on parenteral vaccination methods to thwart systemic disease, but ignored mucosal defenses needed to prevent development of disease (1). To meet these objectives, we introduced the Δ*znuA* mutation, which provided sufficient potency in stimulating host immunity (28, 29), and when combined with Δ*norD* mutation, the double mutant cleared from the host within a sufficient period to facilitate vaccination (30, 31). An oral prime, followed by a nasal boost proved optimal in stimulating CD4^+^ and CD8^+^ TRMs in the lungs, which proved highly effective in resisting pulmonary challenge against virulent wt *Brucella* species (30, 31). A pulmonary challenge model was developed to mimic natural oral and/or nasal exposure to *Brucella* infections seen in livestock and humans (28, 29).

Upon measuring pulmonary T cell responses, B cells were found to be also recruited to the lungs and spleen within 5 days of a single IN delivery of znBM-mC. In contrast, IN wt BM 16M reduced lung and splenic B cells during this same duration of pulmonary infection. Past studies have shown that wt *Brucella* can infect B cells but have suggested that brucellae could not replicate in them (35, 39, 42). Similarly, znBM-mC was also found able to infect B cells. Why B cells are engaged after znBM-mC vaccination remains to be determined, especially in the context that other APCs, specifically, monocyte-derived macrophages, monocytes, and monocyte-derived dendritic cells have been shown to be actively recruited into the lungs shortly after IN znBAZ dosing (46). Initial investigation was conducted to address the potential APC function of recruited B cells. Five days post-IN vaccination, clearly, our evidence showed enhanced activation status of lung B cells notably, the increased CD80, MHCI, MHCII, and CD11b. Infected B cells were found to support the proliferation of CD8^+^ T cells and their stimulation of IFN-γ. Such evidence supports the notion that at least early during the vaccination phase, B cells can also contribute as APCs. Hence, in determining whether other pulmonary infections elicited similar B cell recruitment, it was found that *Mycobacterium tuberculosis* (Mtb) infection of the lungs also showed B cell recruitment and development. Various lung B cell subtypes were described in Mtb-infected humans (47) and mice (48), but these studies focused mostly on their Ab function for Mtb recognition and phagocytosis. However, while Abs did not assist in Mtb clearance, PD-LI^+^ B cell cooperativity with PD-1^+^ CD4^+^ T cells was required to reduce Mtb colonization of the lungs as evidenced by reconstitution of Rag1^-/-^ mice with wt B cells and PD-1^-/-^ T cells (49). The impairment in IFN-γ-producing follicular Th (Tfh) cells in the lungs demonstrated the required cooperativity between B and T cells. Such cooperation between B cells and PD-1^+^ Tfh cells was further validated in CD20-depleted vaccinated macaques challenged with wt Mtb showing reduced capacity to control Mtb infection and reduced Tfh cell presence in lung B cell follicles (49). Such evidence independent of *Brucella* infections suggests that B cells can have Ab-independent function as shown with in our results from the adoptive transfer of naïve or immune B cells into vaccinated µMT mice. Particularly evident was the result of adoptive transfer of immune B cells into naïve µMT mice, which failed to control wt BM 16M colonization of the lungs and spleens.

To assess the role of B cells in protection conferred by znBM-mC, infection studies were performed in both immunocompetent BALB/c mice and immunocompromised µMT mice. Depending on the focus of the experiment, a single IN dose or oral prime- IN boost vaccination strategy was used as a single IN dose was proven sufficient to provide full protection to the lungs of vaccinated BALB/c mice against wt BM 16M challenge. Consistent with what was previously shown (39, 42), µMT mice showed increased resistance to infection noted by the reduced colonization of the lungs and spleens. No significant differences were seen in brucellae colonization of the lungs or spleen in twice nor single znBM-mC-vaccinated µMT mice relative to unvaccinated controls. Despite the lack of protection in µMT mice, studies revealed that the CD4^+^ and CD8^+^ T cell kinetics attributed to znBM-mC vaccination were the same as those found in BALB/c mice. Even in the absence of archetypal structures in lymphoid tissues in B cell-deficient mice (50), greater proinflammatory responses were observed for znBM-mC-vaccinated µMT mice than in similarly vaccinated BALB/c mice, evident by the increased TNF-α^+^ CD4^+^ and IFN-γ^+^ TNF-α^+^ CD4^+^ T cells, and IFN-γ^+^ CD8^+^, TNF-α^+^ CD8^+^, and IFN-γ^+^ TNF-α^+^ CD8^+^ T cells. Thus, an elevated proinflammatory response was indeed induced by znBM-mC vaccination of µMT mice. Examination of the induced resident memory T cells in vaccinated µMT mice revealed that similar magnitude of CD4^+^ and CD8^+^ TRMs, CD8^+^ TEMs, and CD4^+^ and CD8^+^ TCMs were induced relative to vaccinated BALB/c mice. Only a slight, but significant reduction was observed for total CD4^+^ TEMs in vaccinated µMT mice.

Abs are generally considered not to be protective for brucellosis infections (18, 35). Whether Abs are important or not for protection conferred by mucosal znBM-mC vaccination remains to be determined. Perhaps, the recruited B cells in znBM-mC-vaccinated mice may have an alternative role in protection or possibly involved in the elimination of infecting brucellae. Past studies have clearly shown the relevance of cell-mediated immunity to protection without emphasis on the role of B cells (1, 18). Interestingly, the adoptive transfer of either naïve or immune BALB/c B cells into the lungs of znBM-mC-vaccinated µMT mice restored the capacity to reduce lung and splenic colonization by wt BM 16M. Even though the brucellae colonization of these tissues were already reduced because the innate resistance to wt *Brucella* infection, it was notable that the wt BM 16M colonization of both the lungs and spleen were reduced to levels approximating extent of colonization in znBM-mC-vaccinated BALB/c mice. In fact, the adoptive transfer of immune BALB/c B cells into znBM-mC-vaccinated µMT recipients significantly reduced brucellae colonization to nearly undetectable levels in the recipient spleens. Given that donor naïve B cells could also confer immune protection further suggest that an Ab-independent mechanism may be contributing to this enhanced protection in µMT recipients. In the context of vaccination, the T cell response is specific, since no reduction in colonization was observed in naïve µMT recipients adoptively transferred with immune B cells prior to wt BM 16M challenge. In fact, no differences were observed relative to their similarly challenged PBS control µMT mice. The observation that B cells improve protection opposes one study, which showed that adoptive transfer of naïve CD4^+^ T cells together with B cells into Rag1^-/-^ mice lessens the ability of CD4^+^ T cells alone to reduce splenic colonization by wt BM 16M (51). Such B cell inhibition was found to be MHCII-dependent suggesting that B cells were acting as APCs. Alternatively, these B cells could have assumed a regulatory phenotype as suggested by others if these B cells become directly infected by the brucellae (39). While the former study (51) did not vaccinate against brucellosis and only examined immunity induced to wt *Brucella* following infection, the influences of a recall response as done in our study may preempt a direct comparison between these studies. The evaluation performed with wt *Brucella* has the host dealing directly to a number of evasion mechanisms and effector proteins used by *Brucella* to facilitate infection (11, 18). In the present study, znBM-mC vaccination may have already defined the protective epitopes either capable of preventing infection and/or preventing evasion mechanisms taking hold. As such, B cells may be a contributing component to such protection, possibly via uptake of infecting brucellae. Along these lines, in one study found that infection with wt BM 16M of wt or transgenic hen egg lysozyme-specific BCR (MD4) mice resulted in CD19^+^ B cells containing wt BM 16M post-infection (35). Given the differences in Ag specificities, the infection of MD4 B cells is not likely to be mediated via surface Abs. Hence, consistent with our results, the induced B cells by znBM-mC vaccination aid in clearance of challenging wt brucellae.

In summary, the presented results demonstrate a role for B cells in contributing to immune protection when mice are vaccinated with znBM-mC. While the role of Abs has not been completely excluded, our evidence shows B cells serve as APCs for immune T cells. Current studies are evaluating the types of B cells induced by znBM-mC vaccination to better understand their role in immune protection to brucellosis, and whether these memory B cells are capable of infection by wt BM 16M.

## Data Availability

The original contributions presented in the study are included in the article/**Supplementary Material**. Further inquiries can be directed to the corresponding author.
