# Supplementary material for: Co-requirement for B cells to protect against pulmonary wild-type Brucella melitensis challenge following mucosal vaccination with a live Brucella vaccine prototype
Source: Front Immunol. 2026 Oct 23;17:1925725. doi: 10.3389/fimmu.2026.1925725 (PMC13739695; doi:10.3389/fimmu.2026.1925725)
Supplement: Supplementary file 1 [file DataSheet1.docx]

**Supplementary Figure 1**

**Supplementary Figure 1.** Adoptive transfer of naïve B cells into znBMZ-vaccinated µMT mice enables CD4^+^ effector memory T cell (TEM) migration into the lungs and mediastinal lymph nodes (MedLNs). (**A,B**) Groups of µMT mice (n=5/group) were orally dosed with PBS (Group 1) or 10^9^ CFUs znBMZ (Groups 2-6). Groups 3 and 5 were adoptively transferred by the retro-orbital (RO) route with 2-6x10^6^ purified naïve BALB/c B cells on day -1. Groups 1-3 were evaluated for CD4^+^ and CD8^+^ T cells by flow cytometry on day 14 post-vaccination. Additional groups of µMT mice included znBMZ primed on day 0, boosted on day 29 (no adoptive transfer; Group 4); znBMZ primed and boosted with two adoptive transfers (RO day 0 and IN day 28; Group 5); and znBMZ primed and boosted with one IN adoptive transfer on day 28 (Group 6). Groups 4-6 mice were terminated on day 42 post-primary vaccination, and (**C-H**) lungs, (**I-N**) MedLNs, and spleen (**O-T**) were evaluated for T cell subset frequencies: (**C,I,O**) % CD4^+^ T cells, (**D,J,P**) % CD4^+^ TEMs, (**E,K,Q**) % CD4^+^ central memory T cells (TCMs), (**F,L,R**) % CD8^+^ T cells, (**G,M,S**) % CD8^+^ TEMs, and (H,N,T) % CD8^+^ TCMs. A one-way ANOVA and Holm-Sidak method were used for statistical analysis between the indicated groups: ****P < 0.0001, ***P < 0.0005, **P < 0.005, *P < 0.05.
